# Clinical application of 3D-Slicer + 3D printing guide combined with transcranial neuroendoscopic in minimally invasive neurosurgery

**DOI:** 10.1038/s41598-022-24876-1

**Published:** 2022-11-28

**Authors:** Long Zhou, Wenju Wang, Zhiyang Li, Hangyu Wei, Qiang Cai, Qianxue Chen, Zaiming Liu, Hui Ye, Ping Song, Li Cheng, Pan Lei, Sheng Chen

**Affiliations:** 1 Department of Neurosurgery, Renmin Hospital of Wuhan University, No. 238, Jiefang Road, Wuchang District, Wuhan, 430060 China; 2 Department of Critical Care Medicine, Renmin Hospital of Wuhan University, Eastern Campus, Wuhan, China; 3 Department of Neurosurgery, Dangyang Renmin Hospital of Hubei Province, Yichang, China

**Keywords:** Cancer imaging, Cancer therapy, CNS cancer

## Abstract

To explore the clinical advantages of 3D-Slicer + 3D printing guide combined with transcranial neuroendoscopic in minimally invasive neurosurgery. By collecting the datum of patients who underwent craniotomy under 3D-Slicer + 3D printing guide plate positioning combined with transcranial neuroendoscopic in our hospital from October 2021 to February 2022, this paper introduces the accurate planning and positioning lesions of patients before operation and the minimally invasive operation of intraoperative neuroendoscopic and analyses clinical data such as lesion size and surgical bone window size. We collected the case datum of 16 patients who underwent craniocerebral surgery with 3D-Slicer + 3D printing guide combined with transcranial neuroendoscopic, including 5 males and 11 females, aged 46–76 years, including 6 brain tumors (3 meningiomas, 1 glioblastoma, 2 lung cancer brain metastases), 2 cavernous hemangioma, 7 hydrocephalus and 1 chronic subdural hematoma. The lesions of the 16 patients were located accurately before operation and the target areas were reached quickly during operation. Postoperative imaging datum confirmed that the lesions was removed fully, and the ventricular end of shunt tube was in good position. The technology of 3D-Slicer + 3D printing guide plate combined with transcranial neuroendoscopic is not difficult, which has many advantages such as inexpensive equipment, simple operation, easy learning, accurate positioning, and minimally invasive surgery. It is considered to be a practical technology that is feasible, reliable, convenient for diagnosis, preoperative planning and minimally invasive surgery. It is suitable for promotion in neurosurgery and other surgical departments of all medical institutions.

With the progress of medical science and technology and the development of minimally invasive concept, neurosurgery is also developing towards precision and minimally invasive. Its main goal is to remove the lesion to the greatest extent, reduce complications and do not cause new neurological dysfunction. Computer aided medical image processing software (3D-Slicer, ITK-SNAP, etc.) combined with 3D printing technology can truly restore the shape and location of intracranial lesions, which is not only of great help to anatomy-based neurosurgery, but also can accurately locate intracranial lesions through 3D printing guide^1^, effectively avoiding unnecessary nerve damage. In recent years, the creative use of transcranial neuroendoscopic in the operations of intracerebral hemorrhage, intracranial tumors, intracranial aneurysms, arteriovenous malformations, and trigeminal/facial nerve microvascular decompression has brought the minimally invasive surgery of neurosurgery to a new level^2–6^. In this clinical study, through 3D-Slicer preoperative reconstruction of intracranial lesions and accurate preoperative positioning with 3D printing guide, the intracranial lesions can be accurately located, and the most reasonable surgical approach can be planned without intraoperative neuronavigational system. During the operation, transcranial neuroendoscopic minimally invasive surgery is used to clearly show the relationship between the lesions and normal brain tissue, nerves, blood vessels and other structures. It can significantly reduce the surgical trauma and neurological side injury, improve the efficiency and safety of the operation, and reduce the incidence of various complications. Based on skillfully using 3D-Slicer combined with Sina/MosoCam preoperative planning technology, the neurosurgery department of our hospital has newly developed 3D printing technology for preoperative planning and has accumulated some experience in computer-aided medical imaging technology.

## Materials and methods

### General information

In this study, the datum of neurosurgery patients in our hospital of from October 2021 to February 2022 were collected. The inclusion criteria were patients who used 3D slicer reconstruction up the tentorium cerebellum, located through 3D printing guide plate before operation and used neuroendoscopic minimally invasive surgery during operation, including brain tumor, hydrocephalus, cavernous hemangioma, and chronic subdural hematoma. Collect the datum of preoperative MRI + enhancement or head and neck CTA and brain thin-layer CT in the format of DICOM, the three-dimensional pictures reconstructed by 3D slicer before operation, and the datum of 3D positioning guide plate designed and printed before operation. A total of 16 patients with complete datum were collected, including 5 males and 11 females, aged 46–76 years, including 6 brain tumors, 2 cavernous hemangioma, 7 hydrocephalus and 1 chronic subdural hematoma. The lesions of 16 patients were located accurately before operation and found quickly during operation. Postoperative imaging data confirmed that the lesions were removed cleanly, and the ventricular end of shunt tube was in good position. The detailed clinical information is showed in Table 1.

**Table 1:** Clinical data.

No.	Gender	Age	Diagnosis	Lesion size(cm3)	Operation mode	Bone window size (cm^2^)
1	F	66	Right frontal meningioma	2.0*1.3*1.5	Transcranial neuroendoscopic guided excision of frontal lesions	3*3.5
2	F	70	Right temporal lobe meningioma	3*4*4.5	Transcranial neuroendoscopic guided excision of temporal lobe lesions	6*7
3	F	64	Left frontal meningioma	1.7*1.7*1.7	Transcranial neuroendoscopic guided excision of frontal lesions	4*4
4	F	56	Right parietal glioblastoma	4.0*2.9*4.5	Transcranial neuroendoscopic guided excision of parietal lesions	5.5*6.5
5	F	74	Brain metastasis of lung cancer in right thalamus	4*3*2	Transcranial neuroendoscopic guided excision of hypothalamic lesions	3.5*3.5
6	M	69	Brain metastasis of lung cancer in bilateral temporal lobe	2.2*1.8*1.7 and 1.2*0.9*1.0	Transcranial neuroendoscopic guided excision of bilateral temporal lobe lesions	3.5*4.5 and 3*3.5
7	F	70	cavernous hemangioma in right basal ganglia proximal paraventricular	0.6*0.5*0.4	Transcranial neuroendoscopic guided excision of lesions in basal ganglia	3.2*3.2
8	F	75	Cavernous hemangioma of right occipital lobe	0.5*0.5*0.4	Transcranial neuroendoscopic guided resection of occipital lesions	3*4.5
9	F	76	Right frontotemporal chronic subdural hematoma	–	Removal of chronic subdural hematoma guided by transcranial neuroendoscopic	4*4
10	F	64	Communicating hydrocephalus	–	Transcranial neuroendoscopic guided ventriculoperitoneal shunt	–
11	F	53	Communicating hydrocephalus	–	Transcranial neuroendoscopic guided ventriculoperitoneal shunt	–
12	M	74	Communicating hydrocephalus	–	Transcranial neuroendoscopic guided ventriculoperitoneal shunt	–
13	M	46	Communicating hydrocephalus	–	Transcranial neuroendoscopic guided ventriculoperitoneal shunt	–
14	F	51	Communicating hydrocephalus	–	Transcranial neuroendoscopic guided ventriculoperitoneal shunt	–
15	M	54	Communicating hydrocephalus	–	Transcranial neuroendoscopic guided ventriculoperitoneal shunt	–
16	M	59	Communicating hydrocephalus	–	Transcranial neuroendoscopic combined with laparoscopy guided ventriculoperitoneal shunt	–

### 3D-Slicer reconstruction model and design positioning guide

The patient's brain thin-layer CT or CTA, MRI datum were imported into the 3D slicer system in DICOM format (The version 3D Slicer 4.10.2. link: https://slicer.org). Using the above data, reconstruct the tumor, blood vessel, ventricular system, and normal brain tissue, distinguish them with different colors, then reconstruct the scalp model, design the optimal surgical approach according to the relationship between the lesion or ventricular system and the scalp, restore them to the model, and finally print them with a 3D printer (Shenzhen chuangxiang 3D technology Ender-7 3D printer. The version Creality Slicer 4.8.0. link: https://www.crealitycloud.cn/software-firmware/software).

### Preoperative positioning design of surgical incision and intraoperative assistance to guide surgical resection

**Figure 1:**
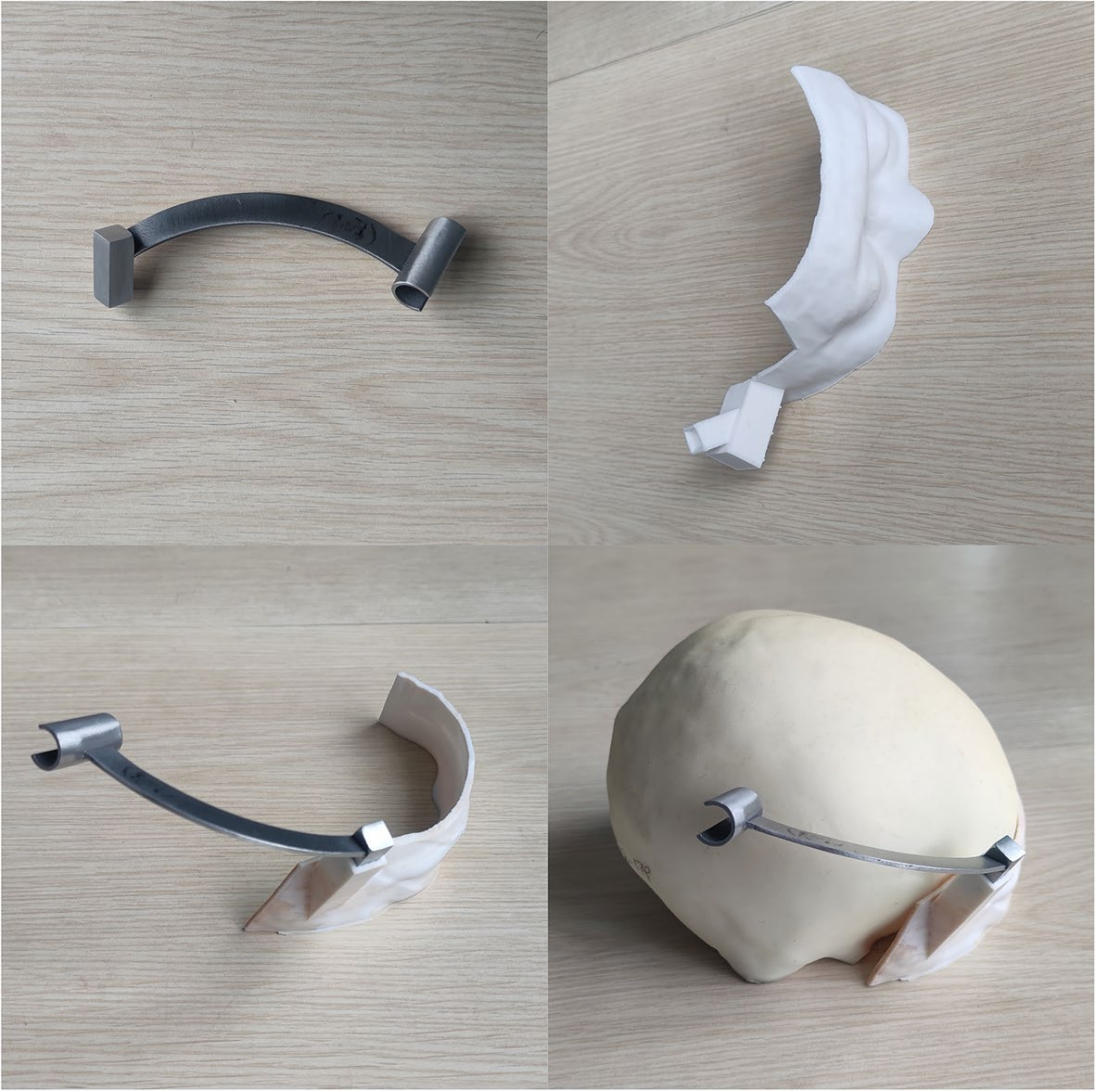
The metal guide plate can be used during operation after disinfection. Combined with the separately designed guide plate, it can guide the direction of intracranial lesions and the puncture direction of ventricular system during operation.

After each patient's preoperative haircut, the printed 3D positioning guide was used to fit the scalp for preoperative scalp positioning and surgical incision design. Through the general matching positioning rod previously designed and printed with metal (which can be disinfected at high temperature and used during operation) (Fig. 1), combined with each individual patient's personalized positioning guide, the direction of deep lesions can be located and guided during operation.

### Ethical approval

All procedures performed in studies involving human participants were in accordance with the ethical standards of the institutional and/or national research committee and with the 1964 Helsinki declaration and its later amendments or comparable ethical standards. This study was approved by the ethics committee of Clinical Research, Renmin Hospital of Wuhan University (WDRY2022-K099), and all the patients and/or their family members signed informed consents.

## Results

### Application of 3D printing positioning guide plate and transcranial neuroendoscopic

The 3D printed lesion model can well reflect the relationship with normal brain tissue. The 3D printed positioning guide plate combined with transcranial neuroendoscopic can make the operation accurate and minimally invasive. The occipital lateral ventricle puncture channel designed according to the reconstructed 3D ventricular system can accurately deliver the ventricular end of the ventriculoperitoneal shunt to the target position of the frontal horn of the lateral ventricle under the guidance of transcranial neuroendoscopic.

### Typical cases

**Figure 2:**
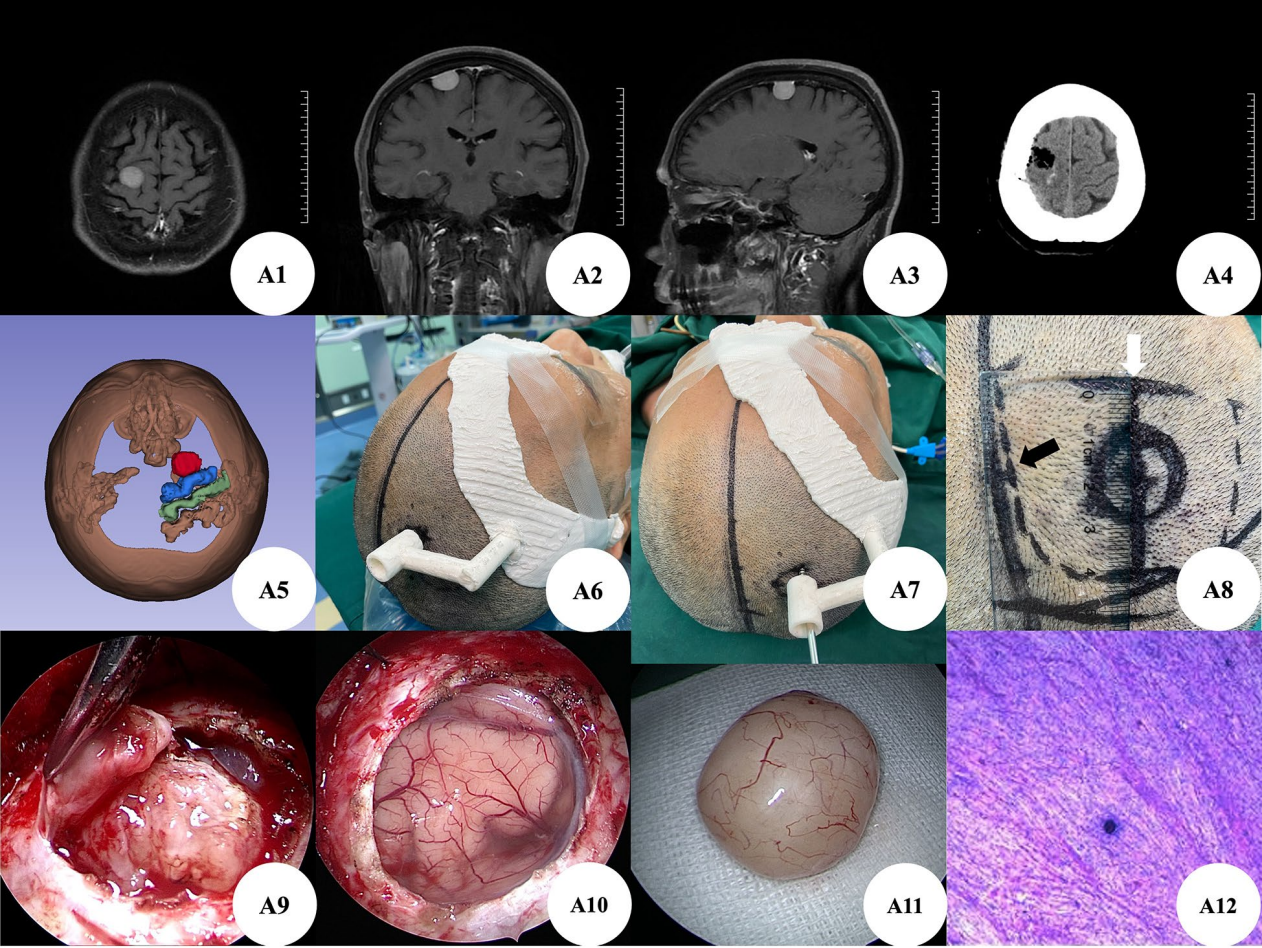
(**A1**–**A3**): Brain MRI enhancement showed right frontal lobe space occupying lesions with uniform enhancement and clear boundary; (**A4**): Postoperative brain CT showed that the intracranial lesions were removed completely; (**A5**): Relationship between intracranial lesions and brain tissue of anterior central gyrus and posterior central gyrus reconstructed by 3D-Slicer; (**A6**–**A7**): 3D printing positioning guide; (**A8**): According to the surgical incision design after 3D positioning guide plate positioning, the circle is the body surface projection of intracranial lesions, the solid line straight incision is the improved small incision, the ruler shows that the surgical incision is about 4 cm, and the dotted line “U” incision is the conventional surgical incision; (**A9**): The lesion can be seen by cutting the dura mater under transcranial neuroendoscopic; (**A10**): The lesion was removed cleanly without obvious side injury to the surrounding tissue under transcranial neuroendoscopic; (**A11**): Completely resected lesions; (**A12**): The postoperative pathological examination.

Case A: The patient was a right frontal meningioma (Fig. 2A1–A3). We reconstructed the location of the tumor and the surrounding sulcus gyrus through the preoperative thin-layer CT data of the patient's brain, which clearly showed that the tumor was in the front of the central anterior gyrus of the frontal lobe (Fig. 2A5). According to the location of the tumor, we designed a positioning guide plate (Fig. 2A6–A7) containing three body surface markers of the nasal root, orbit, and right auricle. Before operation, we accurately located the location of the tumor, and designed a straight incision different from the conventional operation (the conventional operation is a “U” incision) (Fig. 2A8). The tumor was found by cutting the dura mater during the operation (Fig. 2A9), and the tumor was in the middle of the small bone window. It was confirmed that the location was accurate. The tumor was completely removed, and there was no side injury of brain tissue (Fig. 2A10–A11). Postoperative brain CT showed that the tumor was removed completely (Fig. 2A4), and the postoperative pathological examination showed the meningioma (Fig. 2A12).

**Figure 3:**
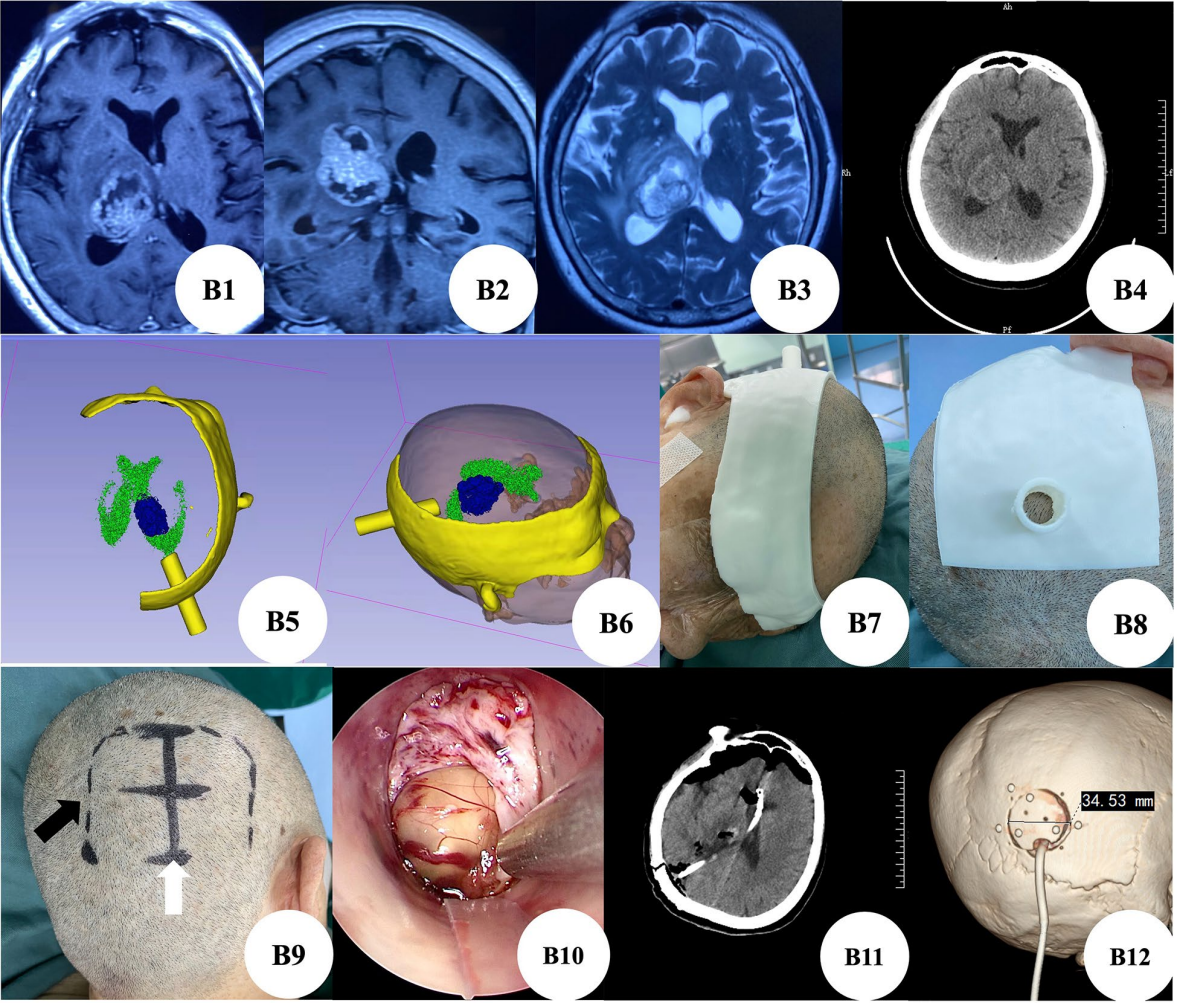
(**B1**–**B3**): Preoperative brain MRI showed space occupying lesions in the right thalamus. (**B4**): Preoperative brain CT showed space occupying lesions in the right thalamus. (**B5**–**B6**): 3D-Slicer reconstructed the relationship between the lesion and lateral ventricle and positioned the three-dimensional image of the guide plate. (**B7**–**B8**): 3D printing positioning guide. **B9**: The straight surgical incision (white arrow) is designed according to the printed positioning guide plate, and the black arrow is the conventional surgical incision. (**B10**): Lesions under transcranial neuroendoscopic. (**B11**): Postoperative brain CT showed that the lesion was resected completely. (**B12**): The three-dimensional reconstruction of the skull showed that the size of the bone window was about 3.5 cm.

Case B: The patient was a brain metastasis of lung cancer in right thalamus (Fig. 3B1–B4). We reconstructed the tumor location and established the guidance channel from the tumor to the scalp through the preoperative thin-layer CT data of the patient's brain, which clearly showed that the tumor was in the right thalamus (Fig. 3B5–B6). According to tumor location, we designed positioning guide plates (Fig. 3B7–B8) and surgical incision (Fig. 3B9). During the operation, the tumor was seen immediately after the dura was cut and the cortical fistula was made along the design direction (Fig. 3B10). The postoperative reexamination of brain CT showed that the tumor was completely removed (Fig. 3B11), the three-dimensional reconstruction of skull showed that the size of bone window was about 3.5 cm (Fig. 3B12).

**Figure 4:**
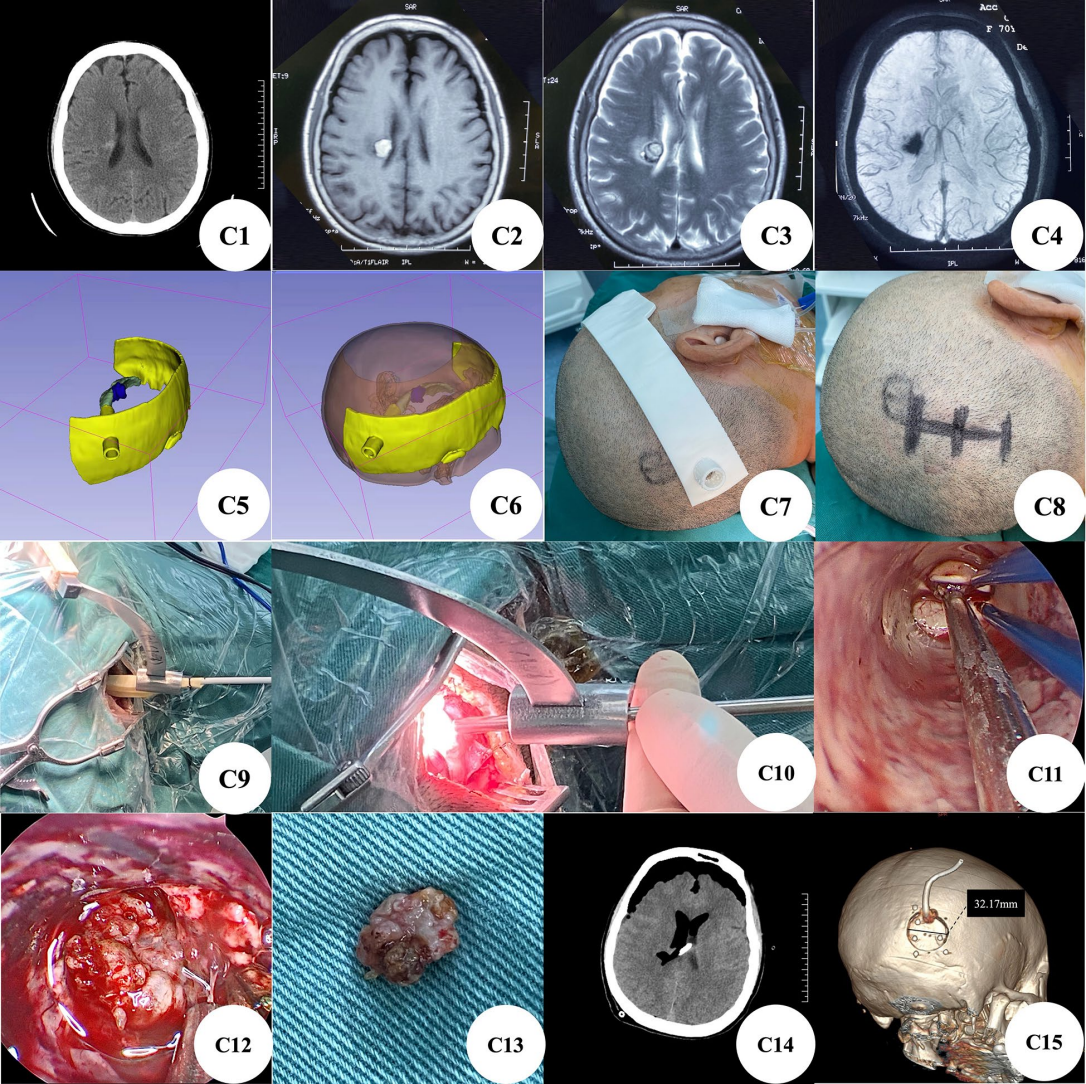
(**C1**): Preoperative brain CT showed high-density lesions in the proximal ventricle of the right basal ganglia; (**C2**–**C4**): Preoperative brain MRI showed lesions in the proximal ventricle of the right basal ganglia; (**C5**–**C6**): 3D-Slicer reconstructed the relationship between the lesion and lateral ventricle, and positioned the three-dimensional image of the guide plate; (**C7**): 3D printing positioning guide; (**C8**): Straight surgical incision designed according to the printed positioning guide; (**C9**–**C10**): Metal 3D guide plate for intraoperative positioning; (**C11**–**C13**): Complete resection of lesions assisted by transcranial neuroendoscopic; (**C14**): Postoperative brain CT showed that the focus was removed completely; (**C15**): The three-dimensional reconstruction of the skull showed that the size of the bone window was about 3.2 cm.

Case C: The patient was a right basal ganglia proximal paraventricular cavernous hemangioma with hemorrhage (Fig. 4C1–C4). We reconstructed the location of the lesion through the preoperative thin-layer CT data of the patient's brain, which clearly showed that the lesion was in the proximal ventricle of the right basal ganglia. We designed a lateral ventricle approach to remove the lesions and established a guiding channel from the lateral ventricle to the scalp according to the above view (Fig. 4C5–C6). According to the location of the lesion, we designed a positioning guide plate (Fig. 4C7) and a surgical incision (Fig. 4C8). The self-made dilator expands the brain tissue to form a channel in the design direction (Fig. 4C9). Transcranial neuroendoscopic enters the lateral ventricle along the design channel for focal resection (Fig. 4C10–C12) and complete resection (Fig. 4C13). After operation, the brain CT showed that the focus was removed completely (Fig. 4C14), the three-dimensional reconstruction of skull showed that the size of bone window was about 3.2 cm (Fig. 4C15).

**Figure 5:**
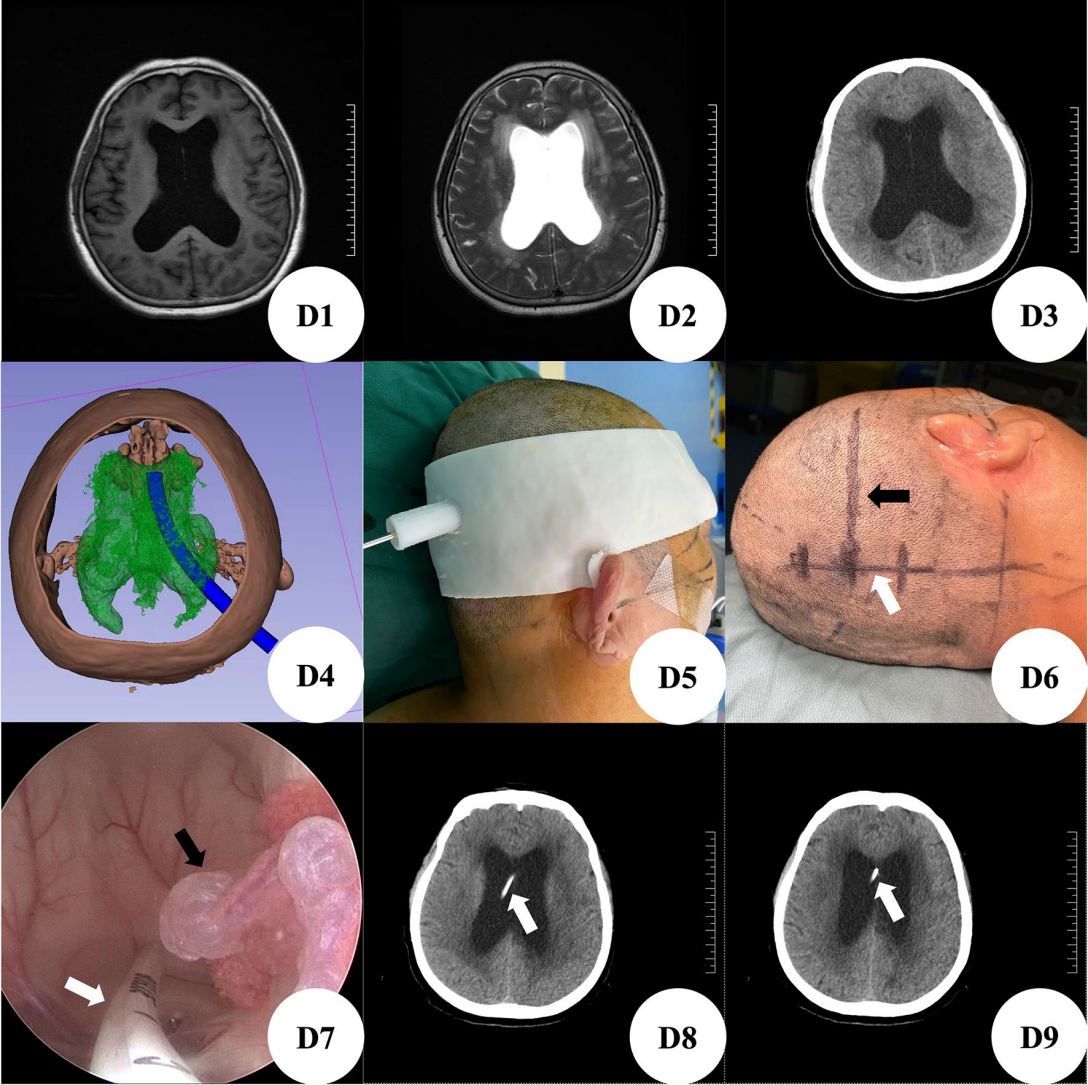
(**D1**–**D2**): Preoperative brain MRI showed hydrocephalus and interstitial edema; D3: Preoperative CT showed hydrocephalus and interstitial edema; (**D4**): 3D-Slicer reconstructed ventricular system and designed puncture channel; (**D5**): 3D printing positioning guide; D6: The straight incision (white arrow) is designed according to the positioning guide plate, and the black arrow is the puncture direction; (**D7**): Choroid plexus and ventricular end of shunt tube from the perspective of transcranial neuroendoscopic; (**D8**–**D9**): Postoperative brain CT showed that the shunt tube was located in the frontal horn of lateral ventricle.

Case D: The patient was a patient with communicating hydrocephalus (Fig. 5D1–D3). We reconstructed the ventricular system and established the guiding channel from the left ventricle to the scalp through the thin-layer CT data of the patient's brain before operation, which clearly showed the expansion of the lateral ventricular system (Fig. 5D4). According to the reconstructed channel, we designed the positioning guide plate (Fig. 5D5). The puncture position(Different from the conventional puncture points of lateral ventricle triangle and occipital angle, namely keen's point, Frazier's point and dandy's point, our team calls them "Cai's point”) was accurately located before operation, and the puncture direction was drawn (Fig. 5D6). During the operation, enter the transcranial neuroendoscopic along the puncture direction to reach the lateral ventricle, and the choroid plexus of the lateral ventricle is clearly visible (Fig. 5D7). It was confirmed that the positioning was accurate. Under the guidance of transcranial neuroendoscopic, the ventricular end of ventriculoperitoneal shunt tube was placed to avoid choroid plexus and reach the frontal angle of lateral ventricle, so as to achieve accurate visual catheterization (Fig. 5D7). Postoperative brain CT showed that the drainage tube was in good position (Fig. 5D8–D9), and the patient's symptoms were relieved significantly.

## Discuss

Accurately finding the lesion in the mysterious brain is the basis for the success of neurosurgery, and the use of transcranial neuroendoscopic is the premise of the implementation of minimally invasive neurosurgery. Computer aided medical image guidance system has been applied to determine the location of focus and surrounding anatomical relationship in neurosurgery and can design the best path to enter the surgical field of vision. With the development of 3D-Slicer and 3D printing technology, 3D printing models have been used to customize anatomical models and medical implants^7,8^, such as plastic titanium plate, peek plate and other repair materials for skull defect repair. Accurate 3D printing model can help neurosurgeons clearly observe the three-dimensional spatial relationship between intracranial lesions and surrounding tissues, help patients and their families better understand the condition of lesions and the risk of surgery, and promote doctor-patient communication. For clinical medical students, accurate 3D printing anatomical model is the most advanced and suitable teaching anatomical model. Previous studies^7,9^ have shown that 3D printed brain model provides good training materials for simulated surgery, contributes to preoperative design, experience accumulation and result verification, effectively improves the surgical effect and reduces surgical complications. Our team has skillfully used 3D-Slicer software for 3D reconstruction and Sina/MosoCam mobile phone app for virtual 3D projection for preoperative planning. In order to overcome the distortion and instability of mobile phone projection, our team further developed 3D printing positioning guide technology on this basis.

In recent years, transcranial neuroendoscopic has developed rapidly and has been widely used in minimally invasive surgery in modern neurosurgery. Transcranial neuroendoscopic is a minimally invasive surgery and a promising alternative to traditional surgery. It has the advantages of good light, multi angle view, close observation and so on, while has great advantages in hypertensive intracerebral hemorrhage surgery^2^, craniotomy aneurysm clipping surgery^3,4^, trigeminal nerve/facial nerve microvascular decompression surgery^6^, and even craniotomy brain tumor surgery. Study^10^ has shown that transcranial neuroendoscopic is more than 90% of hematoma removal in intracerebral hemorrhage surgery. Moreover, the side injury is small, the complications are few, and it is safe and effective. Our previous study^11^ showed that after the fibrin interval of chronic subdural hematoma was removed by transcranial neuroendoscopic minimally invasive surgery, all patients recovered well without recurrence. All drainage tubes were placed accurately without complications. This method has clear vision, less trauma and can reduce the recurrence rate and mortality. The advantages of transcranial neuroendoscopic have been brought into full play in the microvascular decompression of trigeminal nerve/facial nerve. In our previous clinical study^6^, transcranial neuroendoscopic technology can observe the relationship between blood vessels and nerves from multiple angles and explore suspicious compression areas that cannot be observed by microscope. All patients completely used transcranial neuroendoscopic for microvascular decompression and achieved satisfactory results. There were almost no long-term complications except that some patients had vertigo and vomiting in the perioperative period. Of course, transcranial neuroendoscopic also has disadvantages. Transcranial neuroendoscopic is a two-dimensional image, which can’t provide three-dimensional depth perception, and there is the so-called “black under the lamp”, that is, it is impossible to observe the operation area after the endoscope, and side injuries such as brain contusion and laceration may be caused during the operation.

Our current research shows that for the lesions with superficial location, we can achieve accurate preoperative positioning, which changes the previous scalp incision and large bone flap method of using “U” or arc-shaped large incision due to inaccurate preoperative positioning and adopts the skin straight incision to directly locate the lesions, which significantly reduces the surgical trauma. Accurate positioning avoids unnecessary damage to normal brain tissue and nerve fibers and shortens the operation and postoperative rehabilitation time. Case A is a superficial meningioma near the functional area of the central anterior gyrus of the right frontal lobe. Through 3D-Slicer reconstruction and 3D printing guide plate positioning, we accurately locate the tumor location, avoid the harassment of important functional areas such as the central anterior gyrus, and reduce the occurrence of surgical complications. For lesions located in deep or functional areas, we reconstructed the focus through 3D slicer, understood the relationship with the surrounding structure through 3D printing model, designed the most appropriate new surgical approach, accurately located the focus through 3D printing positioning guide plate, and resected the focus using transcranial neuroendoscopic, which achieved accuracy and minimally invasive. Cases B and C are lesions in deep brain functional areas, which have a series of problems such as difficult localization, complex peripheral anatomical structure, and serious consequences if damaged. Similarly, we also used 3D slicer to reconstruct the lesion and 3D printing guide plate to locate the lesion before operation, guided the direction of cortical fistula through the guide plate, accurately and minimally invasive sniped the deep lesion with the help of transcranial neuroendoscopic, completed total resection and protected important functional areas. However, for deep lesions, whether Sina/MosoCam virtual projection or 3D printing positioning technology, the accuracy of intraoperative positioning will inevitably be affected by brain tissue displacement^12^, which is also a problem that needs strict attention in surgery. Case D was communicating hydrocephalus. The expanded ventricular system was reconstructed by 3D slicer. The most appropriate guiding puncture channel was designed according to the shape and deformed ventricular system position, and the appropriate scalp puncture point was marked(According to our experience, this puncture point is different from any previous puncture point. Our team calls it “Cai's point”). During the operation, supplemented by transcranial neuroendoscopic technology, we can avoid the choroid plexus that may block the tube and accurately place the tube to the frontal horn of the lateral ventricle.

## Summary

To sum up, computer-aided medical image processing technology combined with advanced information technology means such as 3D printing operation model and positioning guide plate can play an important role in neurosurgery, while the development of minimally invasive neurosurgery technology such as transcranial neuroendoscopic greatly saves operation time and improves operation quality. The above neurosurgery precise positioning and minimally invasive surgery technology is not difficult. At the same time, it has many advantages, such as inexpensive equipment, simple operation, easy to learn, accurate positioning and minimally invasive surgery. It is a new technology that is practical, reliable, easy to diagnose and preoperative planning, and is suitable for promotion and use in neurosurgery and other surgical departments of all medical institutions.

## Data Availability

All data generated or analyzed during this study are included in this published article and its supplementary information files.
